# The Role of the Microbiome in Driving RA-Related Autoimmunity

**DOI:** 10.3389/fcell.2020.538130

**Published:** 2020-09-29

**Authors:** Cristopher M. Rooney, Kulveer Mankia, Paul Emery

**Affiliations:** ^1^Leeds Institute of Rheumatic and Musculoskeletal Medicine, University of Leeds, Chapel Allerton Hospital, Leeds, United Kingdom; ^2^Musculoskeletal Biomedical Research Unit, Chapel Allerton Hospital, Leeds, United Kingdom

**Keywords:** Initiation of autoimmunity, host-microbiome interaction, gut microbiome, oral microbiome, lung microbiome, rheumatoid arthritis

## Abstract

Once referred to as “normal commensal flora” the human microbiome plays an integral role between health and disease. The host mucosal surface replete with a multitude of immune cells is a vast arena constantly sensing and responding to antigen presentation and microbial by-products. It is this key role that may allow the microbiome to prime or protect the host from autoimmune disease. Rheumatoid arthritis (RA) is a chronic, disabling inflammatory condition characterized by a complex multifactorial etiology. The presence of certain genetic markers has been proven to increase susceptibility to RA however it does not guarantee disease development. Given low concordance rates demonstrated in monozygotic twin studies there is a clear implication for the involvement of external players in RA pathogenesis. Since the historical description of rheumatoid factor, numerous additional autoantibodies have been described in the sera of RA patients. The presence of anti-cyclic citrullinated protein antibody is now a standard test, and is associated with a more severe disease course. Interestingly these antibodies are detectable in patient’s sera long before the clinical signs of RA occur. The production of autoantibodies is driven by the lack of tolerance of the immune system, and how tolerance is broken is a crucial question for understanding RA development. Here we review current literature on the role of the microbiome in RA development including periodontal, gut and lung mucosa, with particular focus on proposed mechanisms of host microbiome interactions. We discuss the use of Mendelian randomization to assign causality to the microbiome and present considerations for future studies.

## Introduction

Complex interactions govern the symbiosis that exists between the human body as the host and our microbiome. It is conceivable to imagine that defense mechanisms capable of continual surveillance for infection but tolerant of our own “healthy microbiome” have evolved (Kamada et al., 2013; Mezouar et al., 2018), allowing the microbiome to prime or protect us from disease. Initial microbiome research focused on descriptions of variant microbiome populations within specific disease states, e.g., diabetes, obesity, inflammatory bowel disease (Tilg and Moschen, 2014; Tralongo et al., 2014; Tomasello et al., 2015; Castaner et al., 2018; Sharma and Tripathi, 2019) or temporal shifts observed over the course of a disease (Kong et al., 2012; Kelly et al., 2016). Autoimmune disorders are a primary group of disorders hypothesized to be triggered by interactions of the microbiome and the host (Chervonsky, 2013). Intriguingly the target tissue which characterizes the clinical syndrome is often distant from the assumed instigating microbial impetus, this scenario is exemplified by rheumatoid arthritis (RA).

As the number of microbiome studies increase and greater details unfold regarding the microbiomes’ physiological role we believe it is important to emphasize two elements of microbiome research. Firstly, strictly speaking the term microbiome refers to all microorganisms and the collection of all their genes and genomes within a microbial community associated with a distinct anatomical region (Lederberg and Mccray, 2001). Therefore this definition would encompass all living organisms at that site, including bacterial, viral, fungal and archaeal populations. This is an important concept as tools for analysis of these complex communities can be divided broadly into functional or taxonomic characterization. Primarily, the former relies on metagenomic shotgun sequencing and the latter on 16S rRNA sequencing but overlap exists, where hypothesized function can be inferred from 16S rRNA sequencing (Ortiz-Estrada et al., 2019) and of course, metagenomics shotgun sequencing is also capable of taxonomic resolution. Taxonomic variation using 16S rRNA often only reports the bacterial portion of the microbiome, albeit the largest and potentially most active microbial component. It is worth noting the fungal, viral and much of the archaeal populations are not reported in 16S rRNA analysis performed. Secondly, it is often cited that the gut microbiome houses up to 10^14^ bacterial cells. While there is agreement that the gut microbiome is indeed the largest human microbiome in terms of cell biomass and genetic potential, the original estimates of bacterial cells outnumbering human cells by 10:1 is likely an over estimation (Sender et al., 2016). Sender et al. (2016) investigated these numbers and found the original estimates stemmed from a single paper published in 1972 (Luckey, 1972) which assumed a homogenous bacterial population density along the alimentary canal, and overestimated the capacity of the human colon thereby amplifying bacterial numbers. Newer estimates suggest a more modest ratio of 1:1 of bacterial to human cell biomass (Sender et al., 2016), however this of course this does not take into account genetic potential and subsequent metabolic pathways which still favor the microbiome as having numerical dominance in terms of transcriptional and subsequent functional activity.

This review article focuses on the mechanistic relationships between the microbiome and the human host and how bacterial perturbations could trigger or potentiate the autoimmune response in RA. Building upon previous review articles (Rosenbaum and Asquith, 2016; Wells et al., 2019; Konig, 2020) we have included a synopsis of the unique immune adaptions at potential offending mucosal sites, focusing on the molecular interactions and a summary of the animal model supporting the mucosal origins hypothesis of RA. We highlight potential new efforts to assign causality to the microbiome using Mendelian randomization on available curated datasets and finally propose some future consideration for further research.

## The Microbiome and RA Autoimmunity

Numerous factors impact the microbiome such as vaginal delivery versus cesarean section in newborns, diet, and antibiotic administration (Hasan and Yang, 2019) with the overriding determinant being anatomical site (Cho and Blaser, 2012). The diversity of a microbiome can be categorized based on which organisms are present, their relative abundance, their temporality and their metabolic activity. Great variation of the microbiota between individuals, and even temporally within the same individual has been demonstrated at the lower taxonomic units (Costello et al., 2009). Each of these streams holds the potential for disease progression or enhancement. For example, the priming of the immune system with a specific antigen exposure or infection with a predominant bacterium and subsequent induction of a pro-inflammatory status could impact disease states.

Rheumatoid arthritis is a chronic, disabling autoimmune disease characterized by a complex multifactorial etiology. The presence of certain genetic markers has been proven to increase susceptibility to RA, however these do not guarantee RA development (Stahl et al., 2010). A 15% concordance has been demonstrated in monozygotic twin studies which clearly indicates a role for environmental factors in disease development (Aho et al., 1986). Furthermore the use of antimicrobials in RA treatment has been established for some time, (Brown et al., 1971) however, the exact mechanism by which they exert their effect is unknown, as is the target group of organisms (Nagra et al., 2019). The production of autoantibodies is driven by the lack of tolerance of the immune system, and how tolerance is broken is a crucial question for understanding RA development. Since the historical description of rheumatoid factor (RF), an IgM antibody directed against IgG, numerous additional autoantibodies have been described in the sera of RA patients. These autoantibodies are directed against various joint components (cartilage, collagen) and non-joint components (enzymes, nuclear proteins, stress proteins) and although are not diagnostically important may contribute to disease by immune complex formation and deposition. The presence of anti-cyclic citrullinated protein antibody (ACPA) is now a standard test for RA, and is associated with a more severe disease course. Interestingly these antibodies are detectable in patient’s sera long before the clinical signs of RA (i.e., clinical arthritis) occur (Nielen et al., 2004). Within this phase of antibody positivity but without arthritis, the joint synovium shows no inflammatory changes (Berglin et al., 2006; Van De Sande et al., 2011). ACPA represent a collection of autoantibodies of various isotypes (IgG, IgM, and IgA) directed against the neutrally charged non-essential amino acid citrulline. The creation of ACPA’s is brought about by a process called citrullination, which is the post-translational modification of arginine, catalyzed by peptidylarginine deiminase (PAD) enzymes resulting in the substitution of arginine to citrulline. This process decreases the proteins ability to form hydrogen bonds due to a lack of positively charged amino acids introducing a structural change within the peptide sequence and subsequent immunogenicity (Kurowska et al., 2017). PAD enzymes are highly conserved throughout evolution, in humans there are five main types of PAD genes (Chavanas et al., 2004) which display both tissue and substrate specificity and are involved in a plethora of physiological functions including tissue structure, apoptosis and immune regulation (Wegner et al., 2010), of note PAD2 and PAD4 are likely more important in RA development given their isolation in the synovium of RA patients (Foulquier et al., 2007). As mentioned ACPA’s may be heterogeneous in terms of their fine specificity to antigens but are highly specific for RA when compared to RF (Kurowska et al., 2017).

The presence of circulating serum ACPA represent a state of systemic autoimmunity, combination of ACPA titers with symptomatology, imaging and major histocompatibility complex (HLA)-DRB1, prediction models can be developed to identify those at the highest risk of progression to RA (Van De Stadt et al., 2013; Rakieh et al., 2015). As previously mentioned, the multifactorial etiology of RA encompasses genetic risk, with the “shared epitope” (SE) accounting for the strongest genetic risk factor (Gregersen et al., 1987; Raychaudhuri et al., 2012; Deane et al., 2017). The SE is located within the third hypervariable region of HLA DRB1 molecule and encodes a five amino acid sequence (Huizinga et al., 2005) with some alleles possessing a higher binding affinity for citrullinated peptides spurring the investigation for a “RA antigen.” The identification of such an antigen is an exciting concept which may provide a malleable risk factor in which RA disease may be moderated or ameliorated. Environmental risk factors are a perpetual interest in RA, as mentioned the use of drugs with antimicrobial properties for the treatment of RA (Ogrendik, 2013) provide a rationale for a possible role of bacteria in RA pathogenesis. Furthermore, infections with specific organisms have been associated with an increased risk of RA development; these include *Porphyromonas gingivalis* (discussed within “Periodontal Microbiome and ACPA” section), *Proteus mirabilis*, *Mycobacterium tuberculosis*, and *Mycoplasma* spp. (Li et al., 2013) the hypothesized mechanism of action includes molecular mimicry and activation of the immune system via a “super-antigen.” Recently gastrointestinal and urogenital infections were shown to be associated with a lower risk developing RA (Sandberg et al., 2015). The authors’ hypothesize that these infections and/or their subsequent treatment with antibiotics may induce a deviation in microbiome composition that has an overall protective effect i.e., disrupting an already high risk microbiome. Smoking status has a long established link with RA as demonstrated by numerous epidemiological studies (Karlson et al., 1999; Criswell et al., 2002; Padyukov et al., 2004; Sugiyama et al., 2010), this combined with the strong link to periodontal disease (PD) (Kasser et al., 1997; Mercado et al., 2001; Pischon et al., 2008; Scher et al., 2012; Äyräväinen et al., 2017) provides clear evidence for the role of the oral microbiome in the development of RA. Continuing along the lines of a microbial impetus for RA, Scher et al. (2013) demonstrated individuals diagnosed with new onset RA had a different microbiome when compared to healthy controls, while Zhang et al. (2015) demonstrated perturbations in both the oral and gut microbiomes could be used to identify those with RA from healthy controls. They also demonstrated that treatment of RA with disease modifying drugs resulted in reversion of the microbiome composition comparable to that of the healthy controls, which undoubtedly is a secondary phenomenon but serves to highlight the interaction between the gut microbiome and joint inflammation. Therefore, understanding the microbiomes role will be paramount in completing this etiological puzzle of RA. In this review we will focus on the mechanisms by which the microbiome may initiate autoimmunity.

## The Niche Environment of the Microbiome

The oral microbiome acts a portal of entry to the human body and therefore has adapted to endure a multitude of physical insults including rapid fluctuations in temperature, pH and external pollutants (air and climate) (Idris et al., 2017). The oral cavity forming the initial segment of the alimentary canal shares many physiological features with the gastrointestinal tract, and indeed the function of gastrointestinal immune cells is far better understood. Distinct to the oral cavity, salivary secretions are a primary component of the oral immune system, and have evolved with the ability to rapidly neutralize microbial threats via the production of large quantities of secretory IgA, mainly in a dimeric form (Brandtzaeg, 2013). The unique properties of the secretary IgA allows it to resist proteolytic cleavage while preventing bacterial aggregation and biofilm formation, a precursor to PD. Stimulus for the production of the IgA comes from inductor sites such as mucosa-associated lymphoid tissue (MALT) (Wu et al., 2014) in particular nasopharynx-associated lymphoid tissue (Brandtzaeg et al., 1999).

**FIGURE 1:**
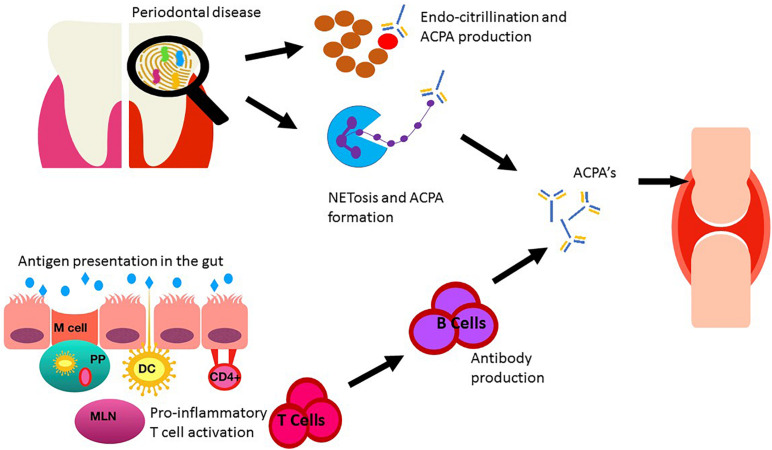
Sent in a different document as requested. Depiction of the role of the oral and gut microbiomes on RA. Oral dysbiosis may increase the burden of citrullination via direct and indirect mechanisms through key pathogens *Porphyromonas gingivalis* and *Aggregatibacter actinomycetemcomitans*. The gut epithelia presents antigens to the underlying immune cells via Microfold cells (M cells) and subsequently to mesenteric lymph nodes (MLN) via dendritic cells (DC) situated in the payer’s patches (PP) within the lamina propria. DC may also sample antigens directly by extension of a dendrite directly in the colonic lumen. Alternative CD4+ T cells may be activated directly by follicular associated enterocytes. This immune activation leads to cytokine production and stimulation of pro-inflammatory T cells (Th17) resulting in B cell activation and antibody production. Anti-cyclic citrullinated protein antibody (ACPA). ACPA’s produced in both the oral cavity and the gut can enter circulation, migrate to the joints and contribute to RA development.

The gastrointestinal (GI) tract is a unique organ providing a pivotal dual role of digestion and maintenance of immune homeostasis. The enormous numbers of antigens presented to the GI tract in the form of food, environmental and microbial antigens are processed at the highly efficient mucosal barrier, which can be divided into three physically distinct elements; the epithelial barrier, the lamina propria and the gut-associated lymphoid tissue (GALT). GALT is further divided into payer’s patches (PP), isolated lymphoid follicles and mesenteric lymph nodes (MLN) which together constitute the largest lymphatic network in the body (Mowat, 2003; Ahluwalia et al., 2017). There are a variety of mechanisms by which the gut is capable of antigen uptake. Briefly antigens can enter via microfold (M) cells and are presented by dendritic cells (DC) to underlying T cells within the PP. Alternatively the DC may enter the lymphatic drainage network and present to next MLN. Direct intraluminal antigen sampling may also occur as DC are capable of extending the dendrite through the epithelial barrier directly into the colonic lumen. Furthermore, follicular associated enterocytes may pass antigens to DC cells or via MHC class II presentation to CD4+ cells (Ahluwalia et al., 2017) see Figure 1. The first line of immune defense relies on a family of receptors known as pattern recognition receptors (PRR’s), these receptors are expressed on innate immune cells and include Toll-Like receptors (TLR) which allow identification of pathogen-associated molecular patterns (PAMPs). Upon sensing a PAMP, TLR enables activation of the immune system and induces subsequent inflammation to eradicate the invading organism (Kumar et al., 2011; Ahluwalia et al., 2017), is it through PAMPs that the mucosal immune system maintains constant surveillance.

Akin to the gastrointestinal system, the mucosal surface of the lung is also exposed to a vast array of antigens, hence the resident immune cells of the bronchial tree must also delineate between potential harmful and innocuous stimuli (Joshua et al., 2017). The lung mucosal barrier in addition to recognition of PAMP’s during pathogen invasion, can also identify damage-associated molecular patterns (DAMP’s) commonly induced by smoking, which may further perpetuate PAMP recognition and subsequent TLR activation (Joshua et al., 2017). Within the lungs, local inflammation fosters the development of inducible bronchus associated lymphoid tissue (iBALT) which is architecturally similar to other non-encapsulated secondary lymphoid structures (PP), having distinct B and T cell elements with supporting APC’s (Carragher et al., 2008). The presence of iBALT therefore confers the mucosal lining of the lungs the ability to generate and potentiate autoantibody production.

## Murine Models of RA

Although the focus of this review is on human studies, it would be remiss not to cover the three main rodent models [collagen induced arthritis (CIA), K/B×N, and SKG] of RA and microbiome research as these studies offer the unique ability for microbial manipulation, and therefore an attractive avenue for mechanistic insights. Within each mucosal section we have included additional important animal studies. Rogier et al. (2017) used a CIA mouse model to investigate the gut microbiome in the pre-RA state. Briefly the CIA murine model is created via immunization of genetically susceptible (DBA/1) mice with type II collagen resulting in a polyarthritis which is both clinically and histologically similar to RA (Brand et al., 2004). Within the preclinical phases of RA a relative decrease in *Bacteroidetes* and *Bacteroidaceae* was identified, while *Firmicutes* and *Proteobacteria* (*Ruminococcaceae*, *Lachnospiraceae*, and *Desulfovibrionaceae*) were increased during the immune-priming phase of arthritis (Rogier et al., 2017). Following elimination of microbial populations (antibiotics) the abundance of intestinal Th17 cells decreased with subsequent attenuation of arthritis. Dynamic temporal microbial changes in this pre-RA phase have also been demonstrated using CIA mouse models (Jubair et al., 2018), with an initial decrease in *Bacteroidetes* and an increase in *Lactobacillaceae*; however, by day 35 of the model (early induction of arthritis at day 14) *Lactobacillaceae* had returned to day 0–7 levels, while *Lachnospiraceae* significantly increased in abundance. The authors found mucosal IL-17A and IL-22 increased on day 14, corresponding to the decreases in *Bacteroidetes* and increases in *Lactobacillaceae* and subsequently normalized on day 35, as bacterial abundances of *Bacteroidetes* and *Lactobacillaceae* returned to day 0 levels (Jubair et al., 2018).

Some *Lactobacillaceae* are known to have a regulatory effect on the immune system, for example *Lactobacillus salivarius* and *Lactobacillus plantarum* transferred to mice prior to the induction of CIA reduced Th17 cells and increased Treg cells resulting in reduced arthritis severity (Liu et al., 2016). As discussed later, *L. salivarius* has been associated with new onset RA in humans. Depletion of microbial load using broad spectrum antibiotics resulted in decreased disease duration and decreased antibody production (Jubair et al., 2018) which was true for both early and late antibiotic administration. Of note, later administration of antibiotics, following the second CIA inoculation, had a greater reduction in disease severity. The anti-inflammatory effect of the reduced microbial load has been attributed to decreased glycosylation of the FC portion of mucosal autoantibodies with subsequent decreased compliment activation leading to decreased inflammation (Jubair et al., 2018). Pfeifle et al. (2017) has shown antibody glycosylation to be an important factor in the transition between asymptomatic autoimmunity and symptomatic disease in RA patients and in CIA and K/B×N models. Tong et al. (2020) demonstrated the microbiome of pre-RA individuals (ACPA positive) was capable of inducing gut mucosal changes in a CIA mouse model. There was significant decreases in Zo-1 gene expression combined with mucosal damage and increases in mucosal Th17 cells, mice inoculated with the pre-RA microbiome displayed earlier and more severe joint disease in comparison to those inoculated with the microbiome from healthy controls (Tong et al., 2020).

The K/B×N mouse model possess a transgenic T-cell receptor and the MHC class II molecule Ag7 resulting in spontaneous arthritis due to the production of autoantibodies against the ubiquitous protein glucose-6-phophate isomerase (Ditzel, 2004). Under germ free conditions, autoimmune arthritis in the K/B×N mice was significantly attenuated, with absence of Th17 cells within the gut mucosa. Inoculation with segmented filamentous bacteria (SFB) reinstated the Th17 cell population and the arthritis phenotype (Wu et al., 2010). In keeping with previous experiments demonstrating colonization with SFB and their ability to induce a proinflammatory state (Ivanov et al., 2009). Thompson et al. (2012) has investigated the taxonomic relationships of SFB and demonstrated those observed in vertebrates to be a distinct linage within the family *clostridiaceae*, suggesting the term Candidatus *Savagella* to be used in future to denote SFB in vertebrates. It is interesting to note that within Thompson et al. (2012) study, SFB observed in arthropods were shown to be a distinct linage within the family *lachnospiraceae*, which has been implicated in the above CIA models. The presence of SFB has been confirmed in the human gut and shown to be associated with increased IgA levels (Chen et al., 2018). Flak et al. (2019) used K/B×N to gain mechanistic insights into the loss of gut homeostasis and showed that arthritic mice had increased gut permeability due to decreased presence of tight junctions and goblet cells when compared to naive mice. They noted downregulation of the gut-protective mediator D5n-3 DPA (RvD5n-3 DPA) and IL-10 expression corresponding with the above physiological changes in the gut mucosal. Following inoculation with *P. gingivalis* further decreases of both RvD5n-3 DPA and Il-10 levels were identified with further disruption of the gut permeability, these changes were reversed upon supplementation with RvD5n-3 DPA restoring gut barrier function, suggesting an additional mechanistic role for *P. gingivalis* in RA development.

A third mouse model; the SKG model [a missense mutation in Zap70 resulting in spontaneous arthritis (Sakaguchi et al., 2003)] has demonstrated induction of arthritis following inoculation of SKG mice with the gut microbiome of early RA patients (Maeda et al., 2016). Germ free SKG mice were either inoculated with pooled feces from healthy controls or early RA patients. A predominance of *Prevotella copri* (discussed later in this review) was identified with within the early RA patients, which was corroborated within the mice receiving the RA microbiome following inoculation and associated with increased Th17 cells and arthritis severity (Maeda et al., 2016). As previous stated, mouse models provide a malleable environment in which to ascertain mechanistic insights into the microbiome’s ability to induce disease, and remain the gold standard of establishing causality in microbiome research (Round and Palm, 2018). However, some caution should be taken given a substantial number of taxa within the human gut fail to colonize the engrafted animal (Zhang et al., 2017), and those that do colonize may foster community structures distinct from the human donor (Staley et al., 2017). Finally, as exemplified by the multifactorial etiology of RA, the ecological factors (diet, lifestyle, genotype) driving the dysbiosis in humans are lacking in the murine models making it difficult to replicate human-microbiome disease associations (Arrieta et al., 2016).

## Periodontal Microbiome and ACPA

Periodontal disease is characterized by environmental risk factors (smoking), genetic risk factors [shared epitope (Marotte et al., 2006)] and persistent inflammation leading to bony destruction, all characteristics shared with RA. We have already highlighted the epidemiological links demonstrating RA patients to have increased prevalence and severity of PD. The fact that PD exists at the time of diagnosis of new RA (Scher et al., 2012; Mankia et al., 2019) suggests it is less likely a consequence of the RA disease process, but possibly an additional target of the disease or a disease initiator. Scher et al. investigated the oral microbiome in new onset RA (NORA) patients prior to disease treatment, and compared it to chronic RA patients and healthy controls. Interestingly there was no difference in the microbiome diversity of the NORA and the other groups, however two taxa, *Prevotella* spp. and *letotrichia* spp. were enriched in NORA patients regardless of PD status. Investigating groups at risk of developing RA allows us to examine disease chronology and investigate disease initiation, before clinical arthritis occurs. This is a unique time point which is unattainable in most disease processes. Mankia et al. demonstrated increased prevalence of PD in ACPA+ at-risk individuals when compared with early RA patients and healthy matched controls (Mankia et al., 2019).

The strongest evidence linking both PD and RA comes in the form of the enzyme PAD. This enzyme has been identified in the organism *P. gingivalis* part of the “red complex” of dental organisms known to cause PD (Mcgraw et al., 1999). Interestingly the genes encoding PAD enzymes in *P. gingivalis* (PPAD) share no sequence homology to human PAD genes and to date *P. gingivalis* is the only bacteria identified containing such genes (Gabarrini et al., 2015). PPAD possesses the same catalytic activity of the human PAD enzymes resulting in citrullination, however PPAD shows preference to the terminal and free arginine residues. This is an important differentiation from human PAD genes (which endocitrullinate) as one of the proposed mechanisms in which *P. gingivalis* induces arthritis is via the release of the virulence factor arginine-gingipanis (RpgB) which cleaves host proteins exposing the terminal arginine residue which is then free to undergo citrullination via PPAD with the creation of neo-epitopes and subsequent ACPA positivity (Wegner et al., 2010). This hypothesis is supported by murine models which demonstrated that mice with laboratory induced arthritis had higher autoantibody production and joint destruction in those infected with wild type PPAD compared to those with mutated deficient PPAD genes (Maresz et al., 2013; Gully et al., 2014; Sandal et al., 2016; Courbon et al., 2019). Sandel et al. demonstrated this pathway was restricted via HLA-DRB1 positive mice suggesting that in addition to the presence of *P. gingivalis* and its associated expressed virulence factors, a genetic predisposition was also required. Remaining on the theme of citrullination, another oral pathogen has been recently identified as a possible etiological agent, *Aggregatibacter actinomycetemcomitans* (Konig et al., 2016). This organism is typically associated with aggressive PD or endocardial disease. Leukotoxin A (LtxA) is a primary virulence toxin produced by *A. actinomycetemcomitans* capable of inducing pore formation into the cell membrane of neutrophils, the resultant transient membrane permeability can then induce dysregulation of human PAD enzymes due to a rapid influx of calcium leading to increased endogenous citrullination, however not shown to be increased in ACPA positive population progressing to RA see Figure 1.

*P. gingivalis* has also been linked to increased production of Th17 cells (Moutsopoulos et al., 2012), a subset of CD4+ cells that have gained much attention in the field of autoimmunity since their identification in Harrington et al. (2005). The primary role of Th17 cells is fighting bacterial (mainly *Staphylococcus aureus*) and fungal (mainly *Candida albicans*) infections (Holland et al., 2007; De Beaucoudrey et al., 2008). Th17 cells are capable of producing a repertoire of pro-inflammatory cytokines IL-17A, IL-17F, and IL-22 (Marwaha et al., 2012), which in turn can result in bone destruction via osteoclastic activation (Sato et al., 2006). Increased levels of IL-17 have been found in the synovium of RA patients and correlate with disease severity (Cascao et al., 2010). Furthermore, CIA mouse models have demonstrated increased Th17 production and large quantities of IL-17 in lymph nodes draining the affected joints in mice infected with *P. gingivalis* PD (Sato et al., 2017; Tsukasaki et al., 2018).

The oral microbiome itself can produce an array of small molecules, some of which share structural homology to self-antigens which may lead to antibody cross reactivity and aberrant targeting of host proteins (i.e., molecular mimicry). Again *P. gingivalis* has been implicated here as its α-enolase protein shares 82% homology to human α-enolase and have been shown to cross-react (Lundberg et al., 2008). Alpha-enolase is consistently identified as an autoantigen (Kinloch et al., 2005) in RA patients. Interestingly higher levels of antibodies against citrullinated α-enolase (anti-CEP-1) were present in individuals, without RA, suffering from chronic PD compared to non PD individuals (De Pablo et al., 2014).

Neutrophil extracellular traps (NETs) primarily act as a defensive mechanisms against invading microorganisms (Kaplan and Radic, 2012). During the process of NETosis the extracellular membrane breaks down allowing the mixing of cellular contents including chromatin and histones proteins with extracellular fluid, creating a matrix capable of affixing invading organisms (Li et al., 2010; Lood et al., 2016). The process of NETosis has recently gained renewed interest in RA (Khandpur et al., 2013), as citrullination of proteins is a key step in NETosis. This process is catalyzed by PAD4 enzymes (Wang et al., 2009) and interestingly individuals suffering from autoimmune conditions display spontaneous NETosis, resulting in the externalization of a number of intracellular molecules which are subsequently recognized as autoantigens (Grayson and Kaplan, 2016; Demoruelle et al., 2017).

Although we have initially stated the microbiome enacts its autoimmune effects via the mucosal surface, which is typically distant to the target tissues, recently both the oral and gut microbiome have been implicated in the translocation of bacteria to the joints. Clinical microbiologists now appreciate that a transient bacteraemia can occur following minor breaks in the mucosal barrier, including brushing ones’ teeth (Maharaj et al., 2012). The possibility of bacterial translocation is increased in the presence of mucosal inflammation such as PD where organisms associated with PD have been identified in the liver and spleen of affected mice (Tsukasaki et al., 2018). Additionally *P. gingivalis* has been shown to persist intracellularly (Li et al., 2008), which may then act as a vehicle to carry this pathogen to joint sites. This hypothesis is supported by the isolation of *P. gingivalis* DNA from the synovium of RA patients (Totaro et al., 2013) suggesting certain bacteria may have a direct inflammatory effect on the joint. If this effect exists it is independent for ACPA as there was no correlation between joint bacteria and ACPA positivity. A summary of possible mechanisms of microbial influence on RA development is provided in Table 1.

**TABLE 1:** Possible mechanism of microbial influence on RA development.

**Study**	**Link to RA**	**Methodology**	**Findings**
Maresz et al., 2013; Gully et al., 2014; Sandal et al., 2016; Courbon et al., 2019	PPAD citrullination	Rodent arthritis model	Infection with *P. gingivalis* aggravated arthritic symptoms
Konig et al., 2016	Citrullination via *A. actinomycetemcomitans*	Mass spectrometry	Neutrophilic hypercitrullination induced by pore forming toxin Lt×A.
Moutsopoulos et al., 2012	Pro-inflammatory cytokine production leading to osteoclastic activation	*In-vitro* cytokine production	Increased Th17 and IL17 production on exposure to *P. gingivalis*
Sato et al., 2017; Tsukasaki et al., 2018	Pro-inflammatory cytokine production leading to osteoclastic activation	Rodent model	Increased Th17 and IL17 production on exposure to *P. gingivalis*
Lundberg et al., 2008	Molecular mimicry	ELISA, mass spectrometry	Cross-reactivity between human α-enolase and *P. gingivalis* α-enolase
Khandpur et al., 2013	Citrullination via NETosis	Neutrophils isolated via sedimentation and quantification via fluorescence microscopy	Increased NETosis in RA patients. NETosis correlated with ACPA presence.
Totaro et al., 2013	Bacterial translocation	PCR	Presence of P. gingivalis DNA in synovial tissue
Taurog et al., 1994	Toll like receptor activation	Rodent model	Arthritis development was dependant on LTR activation by *Lactobacillus bifidus*
Chen et al., 2016	Disease prediction model based on random forest plots.	16S rRNA sequencing in humans. Rodent model used to investigate causality	*Collinsella*, *Eggerthella*, and *Faecalibacterium* identified as predictor organisms of RA. *Collinsella* increase disease severity in rodent models
Kim et al., 2018	Osteoclastic activity inhibited by butyrate	Rodent model	Butyrate inhibited HDAC2 in osteoclast and HDAC8 in T cells. Control of Th17/Treg balance.

## The gut Microbiome and RA

The advent of DNA analyses has expanded our knowledge on the microbiota present within the gut, allowing for the identification of communities but also potential functional pathways. For example, the human microbiome project has produced >70 million 16S rRNA gene sequences (HMP consortium) and together with MetaHIT a European project has suggested great medical significance of the gut microbiome and aimed at developing genomic databases for the enumeration of bacteria. A delicate balance has evolved to create a mutually beneficial relationship between man and microbe representing a symbiosis within the gut. However, we now know alterations in our gut microbiome (dysbiosis) have been linked to a variety of diseases including RA. The change from symbiosis to dysbiosis is a critical transition from microbiome stability to an alternative state, representative of disease. Investigations into the role of the gut microbiome in the development of RA have focused on identifying perturbations in the gut microbiome, thereby resulting in a local mucosal immune dysregulation which may lead to local inflammation and subsequently systemic inflammation.

The gut microbiome has evolved its own unique innate immune homeostasis, with distinct phenotypic expression between gut and blood borne macrophages. CD14 expression is lacking on intestinal macrophages, a key molecule required for the recognition of lipopolysaccharide on bacterial surfaces (Smith et al., 1997). There is also a distinct down regulation of cytokine expression from macrophages isolated from the colon (Smith et al., 2001). Germ-free animal studies have demonstrated a reduced ability of neutrophils to phagocytise and subsequently release superoxide anions into the lysosome (Ohkubo et al., 1990). Innate lymphoid cells capable of helper T (Th) cell regulation via cytokine production play a vital role in immune homeostasis. These cells resemble Th17 cells with respect to their cytokine profile, producing pro-inflammatory interleukins (IL)-17 and IL-22. Additionally, the gut microbiome plays a regulatory role for the adaptive immune response. CD4+ T cells are divided into four major classes depending on their cytokine profile; Th1, Th2, Th17, and T regulatory Cells (Treg) and a careful balance of pro versus anti-inflammatory cells maintain immune homeostasis. Certain bacteria have been associated with the induction of a T cell response, for example *Bacteroides fragilis* induce the differentiation of Treg cells through polysaccharide A present on the bacterial surface resulting in an anti-inflammatory effect that has been shown to have both local mucosal effect but also modulate systemic anti-inflammatory role (Hooper and Macpherson, 2010; Round et al., 2011). Similarly, *Clostridia* spp. (IV and XIVa) have also shown anti-inflammatory effects via IL-10 producing Treg cells after colonization of germ cell mice (Atarashi et al., 2011). The gut microbiome has also been shown to have a pro-inflammatory effect mediated via CD4+ cells differentiation in Th17, this differentiation is induced via the presence of SFB (Ivanov et al., 2009).

Given the interplay described and the multiplicative effect via cytokine activity it is reasonable to assume a pivotal role of the gut microbiome in autoimmune conditions. Germ-free animal studies of HLA B27 positive rats (genetic predisposition) demonstrate that without the input from the gut microbiome peripheral joint disease does not develop (Taurog et al., 1994). Gnotobiotic studies implicate specific bacteria in RA development, moncolonisation with *Lactobacillus bifidus* results in homeostatic imbalance between Treg and Th17 (Abdollahi-Roodsaz et al., 2008). Ivanov et al. (2009) demonstrated a role for SFB specifically *candidatus* spp. resident in the terminal ileum capable to inducting Th17 cell differentiation and subsequent arthritis development.

Biofilm formation within the gut is now receiving more interest as a mechanism of immune dysregulation, tipping the scales between Th17 and Treg cells. Dalpke et al. (2006) described the activation of TLR-9 via bacterial cell contents including DNA, a major constituent of bacterial biofilms (Schlafer et al., 2017). Interestingly biofilm formation has been implicated in the development of systemic lupus erythematosus (SLE), where a structural protein of the biofilm called a curli (amyloid fibril) was capable of binding to bacterial DNA, creating an immunogenic complex inducing an immune response via DC presentation (Gallo et al., 2015). Curli fibers produced by common enteric organisms can activate the NLRP3 inflammasome, leading to the production of IL-1β through caspase 1 and TLR signaling thereby driving a pro-inflammatory response (Rapsinski et al., 2015). However, Curli fibers also have demonstrated the ability to increase epithelia tight junctions (Oppong et al., 2015) via TLR-2 activation and so further work must be done to explore the action of biofilm on autoimmunity. Mucosal epithelial barrier integrity has been linked to RA pathogenesis in mouse models by Chen et al. (2016), who demonstrated colonization with *collinsella* spp. increased mucosal barrier permeability and Th17 cells. Elements known to influence the mucosal barrier integrity include bacteria, their by-products and diet (Horta-Baas et al., 2017; Guerreiro et al., 2018). Microbial by-products particularly short chain fatty acids (SCFA), including acetate, propionate, and butyrate have been linked to amelioration of arthritis development in CIA models (Mizuno et al., 2017; Kim et al., 2018). Mizuno et al. (2017) showed that oral administration of SCFA was able to decrease disease severity by decreasing Th1 cells, and increasing Treg cells thereby damping the immune response.

### The RA Dysbiosis

Using 16S rRNA gene sequencing the microbiome of new onset RA patients, chronic RA patients who are already receiving treatment, established psoriatic arthritis patients and healthy matched controls were analyzed. An overabundance of *P. copri* with new onset RA was found, a predominance of this group was not seen within the microbiome’s of long standing arthritis patients or healthy matched controls (Scher et al., 2013). Functional genomic analysis of the new onset RA patients suggests a decrease in vitamin metabolism (biotin, pyroxidal, and folate) due to a lack of these pathways in *Prevotella* sp. This functional shift may represent a pro-inflammatory environment more resistant to treatment given that folate metabolism is a target for methotrexate, a stable in RA treatment. Interestingly Scher et al. demonstrated an inverse relationship between the abundance of *P. copri* and the presence of HLA-DRB1. The authors’ hypothesize a minimum threshold of *P. copri* is needed to induce disease, which may be lower in those with accompanying genetic risk, although this theory has been recently challenged by Wells et al. (2020), discussed further in the causality section of the review. Using metagenomic shotgun sequencing Zhang et al. demonstrated increased quantities of *lactobacillus* spp. in RA patients at multiple microbiome sites (gut, oral and saliva) which positively correlated with disease activity. *P. copri* abundance was illustrated, within the first year, to be a function of disease duration. Large clustering was identified including *Gordonibacter pamelaeae*, *Clostridium asparagiforme*, *Eggerthella lenta*, and *Lachnospiraceae* bacterium, and smaller clusters containing *Lactobacillus* spp., *Bifidobacterium dentium* and *Ruminococcus lactaris* as over-abundant in the RA microbiome (Zhang et al., 2015).

Recently Alpizar-Rodriguez et al. (2019b) also identified an over-abundance of *P. copri* in individuals at risk of RA, their population included ACPA positive and/or RF positive patients without arthritis comparing them to first degree seronegative relatives. This study demonstrates a dysbiotic microbiome before the onset of clinical arthritis and suggests the gut microbiome may play an active role in RA development. Of note, this study did include individuals with undifferentiated arthritis (likely to include SpA patients) which represents progression of the disease phenotype beyond the pre-arthritis “at-risk” phase (Alpizar-Rodriguez et al., 2019b). Multiple studies describing various dysbiotic bacterial states associated with RA have been published, some demonstrating increased *Prevotellaecae* (Maeda et al., 2016; Jeong et al., 2019; Lee et al., 2019; Rodrigues et al., 2019), some decreased *prevotellaecae* (Vaahtovuo et al., 2008; Breban et al., 2017) and others attributing the dysbiosis to other organisms, such as *Clostridiaceae* (Muñiz Pedrogo et al., 2019) *Blautia*, *Akkermansia*, and *Clostridiales* (Chiang et al., 2019), *lactobacilli* (Liu et al., 2013; Picchianti-Diamanti et al., 2018), *Actinomyces*, *Eggerthella* (Chen et al., 2016; Forbes et al., 2018). Table 2 contains a summary of microbial perturbations found in RA.

**TABLE 2:** Identified microbiome perturbations in RA.

**Study**	**Microbiome**	**Methodology**	**Findings**
Scher et al., 2012	Oral	V1-V2 16S rRNA sequencing	*Prevotella* spp. and *letotrichia* spp. were enriched in NORA
Mankia et al., 2019	Oral	Shotgun metagenomic sequencing	Increased prevalence of periodontitis and *P. gingivalis* in ACPA+ at-risk individuals.
Scher et al., 2013	Gut	V1-V2 16S rRNA sequencing, Shotgun metagenomic sequencing in a subset of patients	Higher abundance of *Prevotella copri* in NORA
Zhang et al., 2015	Oral and Gut	Shotgun metagenomic sequencing	Higher abundance of *Lactobacillus salivarius* in RA patients. *Prevotella copri* was a function of disease duration.
Round et al., 2011	Oral	V4 16S rRNA sequencing	Higher abundance of *Prevotella* spp. in at risk RA population
Rodrigues et al., 2019	Gut	qPCR	Higher abundances of *Bacteroides* spp. and *Prevotella* spp.
Jeong et al., 2019	Gut	Genus specific 16S rRNA PCR	Higher abundances of *Bacteroidales* and *Provotella* in early RA
Maeda et al., 2016	Gut	V5-V616S rRNA sequencing	Higher abundances of Prevotella copri in a subset of RA patients.
Breban et al., 2017	Gut	V3- V416S rRNA sequencing	Lower abundance of *Prevotellaceae* and *Paraprevotellaceae* in RA than healthy controls
Vaahtovuo et al., 2008	Gut	16S rRNA hybridization	Lower abundance of *Bifidobacteria* and bacteria of the Bacteroides-*Porphyromonas-Prevotella* group, *Bacteroides fragilis* subgroup, and *Eubacterium rectale*–*Clostridium coccoides* group compared to fibromyalgia patients
Muñiz Pedrogo et al., 2019	Gut	Shotgun metagenomic sequencing	Higher abundances of *Clostridiaceae* in RA group (arthritis group also contained IBD patients with arthralgia)
Chiang et al., 2019	Gut	V1-V3 16S rRNA sequencing	ACPA-positive patients had higher proportions of *Blautia*, *Akkermansia*, and *Clostridiales* than ACPA-negative patients
Picchianti-Diamanti et al., 2018	Gut	V3-V4 16S rRNA sequencing	Higher abundance of *lactobacillales* in RA. ACPA positivity was indirectly correlated with *Streptococcaceae*, *Erysipelotrichaceae*, *Streptococcus*, *Bacteroides xylanisolvens*, and *Lachnospira pectinoschiza*
Forbes et al., 2018	Gut	V4 16S rRNA sequencing	Higher abundance of *Actinomyces*, *Eggerthella*, *Clostridium III*, *Faecalicoccus*, and *Streptococcus* in all disease cohorts including RA

## Lung Microbiome and RA

Pulmonary disease is a common extra-articular manifestation of RA and may affect multiple respiratory structures including lung parenchyma, pleura, airways and vascular compartments (O’dwyer et al., 2013; Shaw et al., 2015). As previously stated, smoking is a well-established risk factor for RA, and often the presence of respiratory symptoms predates the onset of joint disease. Indeed HRCT has demonstrated underlying lung pathology prior to RA onset (Demoruelle et al., 2012). Furthermore, the presence of autoantibodies in the sputum of seronegative individuals (Willis et al., 2013) suggests the lung may act as a site of autoantibody generation. Scher et al. (2016) demonstrated decreased alpha diversity of the lung microbiome (bronchial alveolar lavage) in individuals with newly diagnosed RA compared to healthy controls. The microbial changes identified by Scher et al. (2016) in this cohort resembled the changes present in the lung microbiome of sarcoidosis patients (granulomatous disease not associated with ACPA positivity); involving decreases in the relative abundance of *Burkholderiaceae*, *Actinomycetaceae, Spirochaetaceae* and the genera *Actinomyces*, *Treponema*, and *Porphyromonas* suggesting a commonality within lung dysbiosis driving inflammation. Of note, there was also a correlation between the presence of the genus *Prevotella* in BAL samples and systemic RF titers and the number of ACPA fine specificities.

While there is a distinct lack of lung microbiome studies in RA or pre-RA, both *Prevotella* and *P. gingivalis* have also been identified within the lung microbiome (Erb-Downward et al., 2011; Goleva et al., 2013) of other inflammatory conditions and therefore it is not impossible to assume they could be present within the RA/Pre-RA population. Both *Streptococcus pneumoniae* and *Streptococcus pyogenes* are capable of causing respiratory infections, similar to *P. gingivalis*, both of these pathogens express α-enolase (Pancholi and Fischetti, 1998; Bergmann et al., 2001) and therefore via molecular mimicry could result in ACPA formation within the lung mucosa. It is interesting to note also the number of respiratory infections has been shown to increase prior to the onset of RA (Arleevskaya et al., 2018) and subside upon RA diagnosis and treatment. Additionally, as smoking induces human PAD genes (PAD2 and PAD4) which increases the overall burden of citrullinated proteins (Kilsgård et al., 2012; Damgaard et al., 2015), it has been demonstrated that citrullinated IL-37 resulted in decreased activity against common respiratory pathogens potentially facilitating bacterial infection within the lungs (Kilsgård et al., 2012). Therefore interplay between lifestyle factors and infections could induce autoimmunity with the lung.

## Causality and the Microbiome

In building the case for the microbiome as a trigger of RA we have relied on several key experimental approaches, specifically aimed at understanding the molecular mechanisms by which the microbiome could trigger autoimmunity. In summary these findings suggest a number of “commensal” organisms capable of inducing ACPA formation, or microbial perturbation resulting in activation and subsequent compensatory immune cascades which could ultimately lead to RA development. Traditional approaches for the assessment of microbial causal relationships required the fulfillment of Koch postulates (Koch, 2010; Duhe, 2011), however these postulates pose a significant hurdle for microbiome studies. Not least of which, the difficulty to culture much of the human microbiome (Browne et al., 2016), furthermore we now appreciate the human host microbiome interactions can be both beneficial or deleterious depending on the organisms abundance and temporality (Cho and Blaser, 2012) which forfeits the major tenants of Koch’s postulates. An alternative approach to help ascribe causality is the use of epidemiology in complex ecosystems like humans, where the putative casual factor is the environment, or in this case, the microbiome. Consideration of the environment as a potential instigator of disease, which may be communicable is integral to the many sights from the field of epidemiology. For example, John Snow considered the father of epidemiology and his description of contaminated drinking water and cholera, to more complex relations of diet and cardiovascular disease or smoking and lung cancer. Epidemiological studies of this nature are expensive and require multiple statistical methods to control for confounders. Furthermore, these latter examples are unpinned by genetics, amongst other factors, and from this perspective microbial perturbations as an impetus from RA would be highly contextual. As we have highlighted earlier in this article, cross-sectional observational studies have demonstrated microbial changes in individuals with RA and pre-RA compared to healthy controls, with animal studies supporting a casual role for the microbiome and RA development. Of course, it is essential to determine the microbiome features that are causal for RA onset from those that are a consequence of the disease process or treatment, and from those that show statistical association due to confounding.

Mendelian randomization (MR) is increasingly used to overcome the multiple confounding effects of observational studies and has been used as an alternative method to ascribe causality (Sekula et al., 2016; Davies et al., 2018). MR relies on the presence of naturally occurring genetic variants within our human genome, and assigns these as instrumental variables to investigate the effects of a modifiable risk factor (the microbiome) on a disease outcome (RA). Valid genetic instrumental variables for RA studies must fulfill three assumptions; the selected gene must be associated with the microbiome (relevance assumption), must not be associated with any related confounders (independence assumption), and does not affect RA except through the microbiome (exclusion restriction assumption) (Davies et al., 2018). The nature of genetic inheritance which allows the use of randomization introduced during meiosis and fixed at conception allows genetic variants to act as plausible instrumental variables. Inamo has used MR to investigate for a causal relationship between the gut microbiome and RA (Inamo, 2019). Utilizing two datasets from genome-wide association studies; one for the gut microbiome as the exposure and another for RA as the outcome, 26 single nucleotide polymorphisms (SNP) were identified to be associated with decreased gut microbial diversity. One SNP (rs1230666) was found to be independently associated with RA development and therefore removed, following its removal the remaining 25 SNP’s failed to demonstrate the microbiome as a casual factor for RA development (Inamo, 2019), suggesting the observed microbial dysbiosis in RA is a secondary phenomenon. However, Alpizar-Rodriguez et al. (2019a) has highlighted that microbial dysbiosis does not always equate to decreases in microbial taxa, and rather is it a compositional shift of the microbiome that is driving the RA dysbiosis (Kishikawa et al., 2020). Therefore MR analysis based a selection of SNP’s that are known to affect the gut microbiome by decreasing the numbers of taxa present within the gut is unlikely to yield a casual effect, given an arbitrary decrease of taxa is in contradiction to the current observational studies.

Using the same datasets as Inamo, lee again used MR to question the causal effects of the microbiome on RA (Lee, 2020). In this study 32 SNPs were identified as instrumental variables, as opposed to original 26 identified by Inamo, presumably by including SNP’s known to alter bacterial taxa in either direction, not limiting to those that decrease bacterial taxa only. Following removal of rs1230666 SNP, known to independently influence RA development, the MR analysis did show significance and supported a casual role for the microbiome in RA development. The use of MR to assign causality to the gut microbiome obviously relies upon the selected SNPs and the strength of their influence on the microbiome, Rothschild et al. (2018) has demonstrated that genetics have a poor effect on microbiome composition. However, a recent study by Wells et al. (2020) has demonstrated that a polygenic risk score for RA was positively associated with the presence of *Prevotella* spp. within the gut microbiome of non-RA patients, they went on to validate their findings within an independent cohort composed of first degree relatives of RA patients carrying the HLA DRB1 risk allele. An inverse relationship between *P. copri* and HLA DRB1 has previously been reported (Scher et al., 2013), here the authors’ suggest the differences identified are likely a result of population characteristics or unmeasured confounding (Wells et al., 2020). They also found an association between individuals with preclinical RA and *P. copri*.

Interestingly, there was no association between the polygenic risk score and overall microbial diversity (Wells et al., 2020), again adding weight to the theory that MR analysis based on decreased taxa is unlikely to be an accurate measure of RA dysbiosis.

## Future Considerations

There is much interest in the microbiome community to move past the traditional taxonomic description of the microbiome to understanding its functional capacity, which has mainly relied upon metagenomic sequencing which is expensive [although methods to infer function from 16S rRNA are available (Ortiz-Estrada et al., 2019)]. Recently Hillmann et al. (2018) described shallow shotgun sequencing, allowing better taxonomic resolution and functional analysis for a fraction of the cost of whole metagenome shotgun sequencing, although if wishing to identify the rarer taxa which may delineate a disease phenotype then depending on research budget 16S rRNA may still be the better option. It is worth pointing out that some RA studies have relied upon qPCR to identify microbes of interest, which obviously identifies only the organisms carrying the reciprocal primer target sequence, which might create a skewed view of the microbiome in these individuals; it is possible that in the future qPCR might be the technology that is taken to the clinic if a unique microbiome signature indicative of RA disease was identified, but we are not at that stage yet. It has been over 10 years since the first publication of the human microbiome project (Turnbaugh et al., 2007), which has been consolidated by large phenotyped cross-sectional population studies (Falony et al., 2016; Zhernakova et al., 2016). These studies highlight the importance to account for multiple covariates into microbiome research such as diet, gut transit time, medications and intrinsic factors, to date no such RA study has included these important factors and this is a key point that needs to be addressed moving forward.

As we have highlighted in this review mucosal surfaces including oral, gut and lung microbiomes can play an important role in initiating and perpetuating inflammation. It appears that induction of autoimmunity may not occur at a single site but in fact these microbiomes may be acting in tandem creating a systemic multiplicative effect. A recent study by Schmidt et al. (2019) demonstrated that extensive transmission of bacterial species exists between the oral and gut mucosal sites, demonstrating >40% of bacterial species were prevalent (>10%) in both gut and salivary samples in a population including RA patients. Taken into account future microbiome studies should investigate a homogenous at-risk population, incorporate known factors effecting the microbiome, examine multiple mucosal sites, and ideally provide a longitudinal prospective as individuals progress toward the disease phenotype.

## Author Contributions

CR initially drafted the manuscript. KM and PE critical revising of the manuscript. All authors contributed to the article and approved the submitted version.

## Conflict of Interest

PE has provided expert advice for Abbvie, Pfizer, Amgen, Schering-Plough, Roche, BMS, Novartis, Lilly, Gilead, and Genentech. The remaining authors declare that the research was conducted in the absence of any commercial or financial relationships that could be construed as a potential conflict of interest.
